# Emergency laparoscopic splenectomy for torsion of wandering spleen in a geriatric patient: A case report

**DOI:** 10.1016/j.ijscr.2019.07.021

**Published:** 2019-07-19

**Authors:** Novia Ayuning Nastiti, Muhammad S. Niam, Phong Jhiew Khoo

**Affiliations:** a University of Brawijaya, Malang, 65145, East Java, Indonesia; b Newcastle University Medicine Malaysia, Iskandar Puteri, 79200, Johor, Malaysia

**Keywords:** Wandering spleen, Geriatric, Torsion, Acute abdomen, Splenectomy, Laparoscopic

## Abstract

**Introduction:**

Wandering spleen (WS) is an uncommon congenital or acquired condition where the spleen is displaced from its normal position at the left hypochondrium to anywhere within the abdominal or pelvic cavity. The incidence is extremely rare in the geriatric population.

**Presentation of Case:**

We present a rare case of WS torsion in a 69-year-old elderly patient who presented with an acute abdomen. Physical examination revealed a tender right lower quadrant abdominal mass. Imaging studies confirmed the diagnosis of WS torsion with features of infarction. Subsequently, an emergency laparoscopic splenectomy was performed.

**Discussion:**

A WS occurs due to the hypermobility of the spleen secondary to the absence or laxity of splenic suspensory ligaments. It is more commonly seen in children and adults in the third decade of life. Symptoms are usually attributed to the consequences of splenic vascular pedicle torsion. Exhibited symptoms might be unspecific; thus, radiological modalities are essential to determine the diagnosis and aid in planning its management. The treatment of choice is either open or laparoscopic splenopexy or splenectomy.

**Conclusion:**

Due to potentially life-threatening consequences and the rarity of such cases, a thorough history, detailed physical examination, and objective investigation are the pillars to attain a prompt diagnosis for appropriate management to be conducted as soon as possible to minimise complications.

## Highlights

• Wandering spleen is rare in geriatric / elderly population.
• Wandering spleen torsion may present as acute abdomen.
• Imaging is needed to diagnose wandering spleen.
• Imaging is essential in assessing splenic perfusion in case of torsion.
• Non-viable spleen can be managed by laparoscopic splenectomy.

## 1 Introduction

Wandering spleen (WS) is a rare congenital or acquired condition where the spleen is not found in the left hypochondrium [[1], [2], [3], [4], [5], [6], [7], [8], [9], [10], [11]]. This abnormality occurs secondary to the absence or laxity of splenic suspensory ligaments [[1], [2], [3], [4], [5], [6], [7], [8], [9], [10], [11], [12], [13]]. The spleen might be displaced within the abdominal or pelvic cavity due to hypermobility [2,[6], [7], [8], [9], [10], [11]]. Most patients presenting with WS are children or adults in the third decade of life [1,4,6,9,10]. The incidence is rare in the geriatric population [5]. The most common complication of WS is torsion of the splenic vascular pedicle, which could lead to splenic infarction and rupture [1,2,10,13]. Clinical presentations vary from asymptomatic incidental discovery to a life-threatening acute abdomen [1,2,8,10,11]. Imaging modalities can aid in diagnosis and assessment [[1], [2], [3][5], [6], [7], [8], [9], [10][12], [13], [14]]. A WS is managed surgically with a splenopexy or splenectomy, using a laparotomy or laparoscopic approach [[1], [2], [3], [4],[6], [7], [8], [9], [10], [11], [12], [13], [14]]. We present a rare case of WS torsion in an elderly patient who presented with an acute abdomen, and an emergency laparoscopic splenectomy was performed. This case report is reported in line with SCARE criteria [15].

## 2 Presentation of case

We present the case of a 69-year-old female with WS torsion. She has complained of intermittent right lower quadrant abdominal pain since a month ago, and the pain worsened over the previous five days. She did not feel feverish and denied any gastrointestinal symptoms. She did not experience symptoms of urinary tract infection. No family history of connective tissue disease or malignancy was identified. She bore to two children three decades ago via vaginal deliveries. Last year, she underwent an open cystectomy for a symptomatic functional ovarian cyst.

Upon examination, we noted an intra-abdominal mass at the right lower quadrant of the abdomen, measuring roughly 10 cm × 7 cm. The smooth-surfaced mass was mobile, firm, and tender upon palpation. Other physical examinations were normal.

**Fig. 1:**
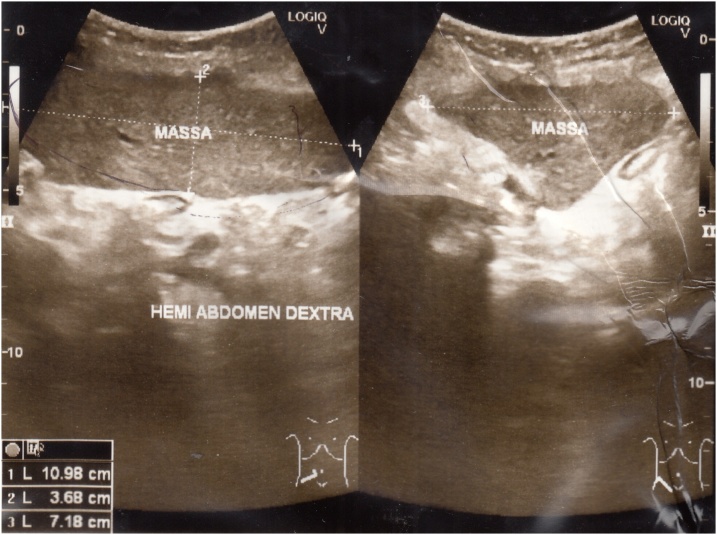
Ultrasonography of the abdomen showing a heterogeneous mass within the right abdominal cavity.

**Fig. 2:**
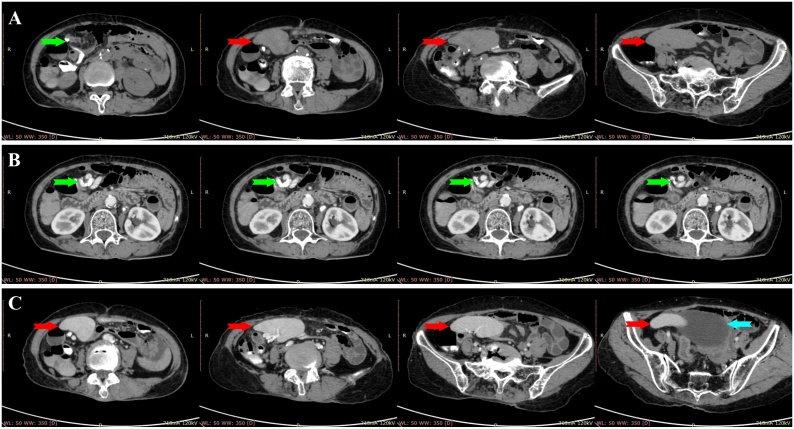
(A): Axial view of the abdominal non-contrasted CT scan showing a suspicious pedicle (green arrow) and an ectopic spleen (red arrow) in the right abdominal and pelvic cavities. (B): Axial view of the abdominal contrasted CT scan showing a twisted splenic vascular pedicle (green arrow) in a whirlpool disposition. (C): Axial view of the abdominal contrasted CT scan showing an enlarged ectopic spleen (red arrow) extending from the right abdominal cavity to the pelvic cavity, lateral to the urinary bladder (blue arrow).

**Fig. 3:**
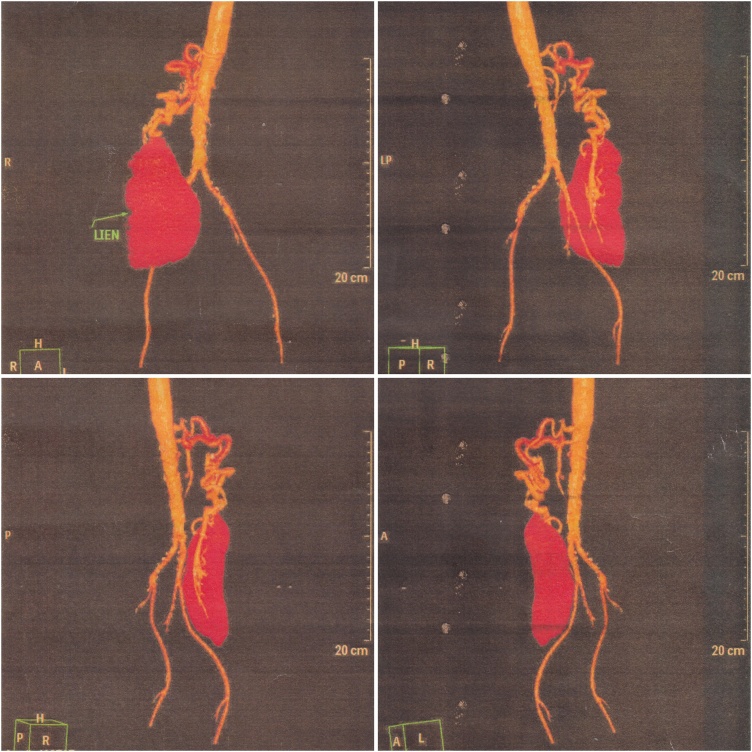
Volume-rendered technique CT abdominal angiography showing an anticlockwise torsion of the elongated splenic vascular pedicle in a whirlpool disposition.

We proceeded with an abdominal ultrasonography, revealing a heterogeneous mass within the right abdominal cavity and absence of the spleen in its normal position (Fig. 1). The features pointed towards a WS. Thus, a contrast-enhanced computed tomographic (CT) scan of the abdomen was ordered, revealing a 12.4 cm × 3.5 cm × 7.4 cm ectopic spleen in the abdominal cavity occupying the right lumbar, iliac, and pelvic regions (Fig. 2). Subsequently, an abdominal CT angiography was performed, which demonstrated anticlockwise torsion of the elongated splenic vascular pedicle in a whirlpool disposition with splenic infarction characteristics (Fig. 3). Laboratory investigations of the patient were normal.

**Fig. 4:**
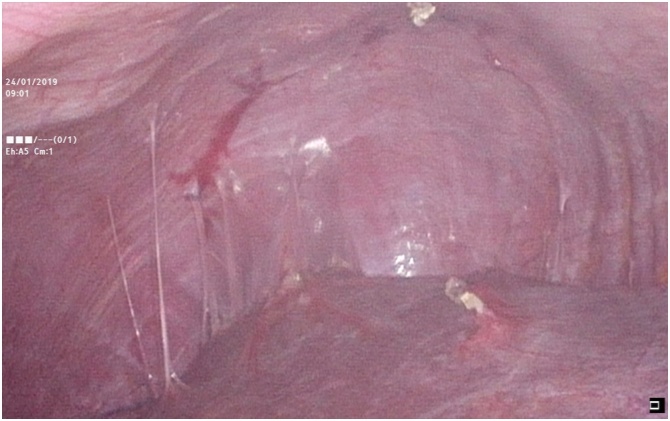
Image from video records made during laparoscopy revealing the absence of the spleen in its normal position in the left hypochondrium.

**Fig. 5:**
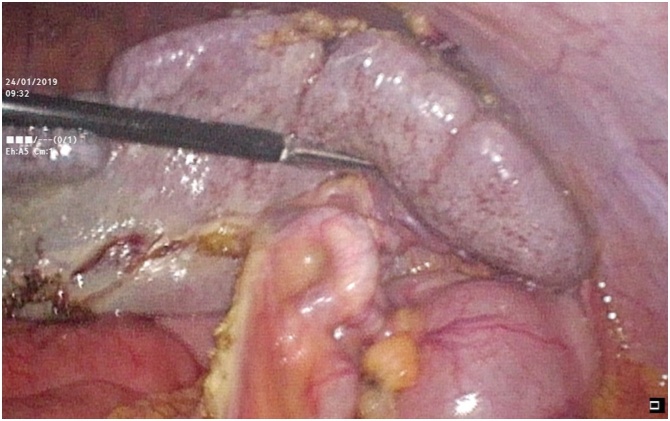
Image from video records made during laparoscopy revealing a non-viable spleen after adhesiolysis and detorsion in the right iliac fossa.

Soon after, an emergency exploratory laparoscopy was planned for the patient. Intra-operatively, the enlarged spleen was found in the right iliac fossa, displaced from the left hypochondrium (Fig. 4). The ptotic spleen had twisted anticlockwise around its elongated vascular pedicle and had no ligamentous attachments. Peri-splenic adhesions to the anterior abdominal wall were found. After adhesiolysis and detorsion, the spleen was deemed non-viable (Fig. 5). Due to the intra-operative findings, a laparoscopic splenectomy was performed. From the subsequent histopathological examination of the spleen, extensive infarctions were inferred. The patient had an uneventful recovery. Vaccines including pneumococcal, meningococcal, and *Haemophilus* influenza type b were given to the patient post-operatively.

## 3 Discussion

A WS is also known as a displaced, ectopic, pelvic, or ptotic spleen [1,4,6,7,10,13,16]. Limited literature was found regarding this uncommon clinical entity, most including case reports and series [2]. Only around 500 cases of WS, which presented with abdominal pain, were reported [1,5,7]. The incidence rate of WS is 0.05%–0.5% [7,9,10,13]. The majority of the patients presenting with WS are children under 10 years old, especially those under 1 year old and young adults 20–40 years old, predominantly fertile females [1,4,6,9,10]. Statistically, WS is 6–7 times more common in females than males above 10 years old, but interestingly, there is a male predominance for those under 1 year old with the ratio of 2.5:1 [1,6,[9], [10], [11]]. No gender predominance exists in the group comprising 1- to 10-year-olds [6]. The incidence is rare in the elderly population [5]. McFee et al. [5] discovered only 13 reported cases of WS with patients aged above 61 years, whereas our review yielded only three cases of WS involving older patients, of which only two were geriatric patients (> 65 years) and the other patient is a 64-year-old man [2,5,9]. Of the three case reports, one patient had thalassemia with splenomegaly on presentation [9]. In our case, the patient is a 69-year-old elderly woman without any co-morbidity.

The pathophysiology of WS is characterised by the hypermobility of the spleen secondary to the absence or excessive laxity of the following primary splenic suspensory ligaments: gastrosplenic, splenorenal, splenocolic, splenophrenic, pancreaticosplenic, and pre-splenic folds [1,4,[6], [7], [8], [9], [10],^13^,^16^]. Due to the abnormality or absence of these ligaments, the splenic vascular pedicle in WS is susceptible to elongation and torsion [1,7,8,12]. The aetiology of WS is considered to be multifactorial and broadly divided into congenital anomalies and acquired conditions [1,6,7]. Congenital WS is secondary to the failure of fusion or incomplete fusion between the dorsal mesogastrium and posterior abdominal wall during foetal development [1,3,6,[8], [9], [10], [11]]. Acquired WS might be due to trauma or other underlying conditions, such as multiparity, splenomegaly, connective tissue diseases, and enlargement or absence of a kidney [1,2,[6], [7], [8], [9],^11^]. Hormonal changes during pregnancy contribute to the increased laxity of the ligaments, which could explain the higher prevalence of WS in fertile females [7,8,10,11]. Our patient had two previous pregnancies, which might be a contributing factor.

Most patients with WS are asymptomatic [10]. Commonly, WS is detected incidentally in physical examination as an abdominal mass or in imaging studies performed for other conditions [3,[6], [7], [8],[12], [13], [14]]. Paediatric WS patients usually present with acute abdominal pain, whereas abdominal mass is the most common complaint in adult WS patients [6]. Clinical features of WS are usually attributed to the consequences of splenic vascular pedicle torsion [1,3,8,10]. Initially, the abdominal pain might be intermittent due to splenic congestion with recurrent torsion and spontaneous detorsion of the splenic vascular pedicle, but the patient might develop an acute abdomen secondary to acute torsion with splenic infarction or rupture [3,[7], [8], [9],^11^,^13^]. Chronic torsion of the splenic vascular pedicle and splenic sequestration resulted in splenomegaly and presented as an abdominal mass [6,8,10]. Other reported unspecific symptoms are nausea, vomiting, and fever [2,6,13]. Recurrent acute pancreatitis is a rare presentation and complication of WS, where the tail of the pancreas is twisted along with the splenic vascular pedicle at the splenic hilum, causing pancreatic inflammation [2,3,11]. Laboratory investigations might reveal thrombocytopenia or Howell-Jolly bodies in some patients due to sequestration, hypersplenism, or functional asplenia, but otherwise are non-specific in diagnosing WS [1,3,5,6,10]. Our patient experienced intermittent abdominal pain prior to presenting an acute abdomen, and her laboratory investigations were not significant.

Due to the non-pathognomonic history and clinical manifestation, radiological modalities are paramount in attaining a definitive diagnosis [1,3,5,6,[9], [10], [11], [12], [13], [14]]. Imaging studies provide vital information, such as splenic blood flow and viability to aid in management planning [3,7,11]. Commonly, ultrasonography and CT scans are performed to aid in assessing and diagnosing WS [2,3,5,[7], [8], [9], [10],^14^]. Other radiological studies, such as X-ray, barium studies, nuclear scintigraphy, and magnetic resonance imaging, have been reported [1,6,10,12,13]. The abdominal ultrasonography demonstrates an abnormal intra-abdominal or pelvic mass with concomitant absence of the spleen in the left hypochondrium [3,5,[7], [8], [9],^14^]. A corresponding Doppler or duplex study can be done to evaluate the splenic blood flow [3,[7], [8], [9]]. A contrast-enhanced CT scan and CT angiography can precisely locate and delineate the ectopic spleen, provide information on splenic perfusion, and reveal any whorled splenic vascular pedicle indicating torsion [4,5,9,13,14]. Torsion usually occurs in a clockwise configuration [2,9]. Compromised splenic blood flow and heterogeneous echogenicity of the spleen are features of splenic infarction [7,8]. In our case, an abdominal ultrasonography, contrast-enhanced CT scan, and CT angiography were performed, which demonstrated a heterogeneous mass in the right abdominal cavity, suggestive of WS torsion with splenic infarction. Interestingly, the torsion showed an anticlockwise rotation on the CT scan, which was also observed intra-operatively.

Moreover, 65% of asymptomatic WS patients treated conservatively developed complications, such as torsion, compression of other organs, and susceptibility of the spleen to trauma; thus, non-operative management is inadvisable [1,6,7]. A WS only accounted for 0.1%–2% of all splenectomies [1,2,4,5,[9], [10], [11],^14^]. Splenectomy is recommended for a non-viable spleen, splenic infarction or rupture, thrombosis, and splenomegaly [3,[6], [7], [8], [9]^12^]. Otherwise, splenic function preservation with splenopexy is preferred in uncomplicated WSs to avoid risk of an overwhelming post-splenectomy infection, especially in young patients [2,3,6,8,14]. Laparoscopic approaches are considered more ideal compared to laparotomies because they are less painful and have better cosmetics, lower wound complications, shorter hospitalisation, lesser morbidity, and earlier return to normal activity [1,8,17]. A laparoscopic approach is technically feasible because WS is relatively free from attachments and other organs [1]. However, Benevento et al. [17] discovered only five reported cases of a laparoscopic approach to WS, whereas our review yielded one case of laparoscopic splenectomy for WS [14]. In our case, an emergency laparoscopy was offered, and we decided on splenectomy, as the spleen was deemed non-viable even after detorsion. Intraoperatively, peri-splenic adhesions were encountered, possibly from recurrent ischaemic events of the spleen.

## 4 Conclusion

Ultimately, due to potentially life-threatening consequences and the rarity of such cases, a thorough history, detailed physical examination, and objective investigation are the pillars to attain a prompt diagnosis of WS for appropriate management to be conducted as soon as possible to minimise complications.

## Funding

No source of funding

## Ethical approval

Ethical approval has been exempted by our institution as this publication is a case report and not a randomized trial or a case series, provided that the patient gave her written consent both for operation and the publication of this case.

## Consent

Written informed consent was obtained from the patient for publication of this case report and accompanying images. A copy of the written consent is available for review by the Editor-in-Chief of this journal on request.

## Author contribution

Novia A. Nastiti is the first author of this paper. Muhammad S. Niam is the surgeon in charge. Novia A. Nastiti and Muhammad S. Niam were involved in the surgery. Phong Jhiew Khoo is the corresponding author and performed critical revision to this paper. Novia A. Nastiti, Muhammad S. Niam, and Phong Jhiew Khoo participated in the writing of this paper. All authors read and approved the final manuscript.

## Registration of research studies

Not applicable.

## Guarantor

Novia A. Nastiti

Muhammad S. Niam

Phong Jhiew Khoo

## Provenance and peer review

Not commissioned, externally peer-reviewed.

## Declaration of Competing Interest

No conflicts of interest.
